# Tracking and visualization of space-time activities for a micro-scale flu transmission study

**DOI:** 10.1186/1476-072X-12-6

**Published:** 2013-02-07

**Authors:** Feng Qi, Fei Du

**Affiliations:** 1 School of Environmental and Life Sciences, Kean University, 1000 Morris Ave., Union, NJ, 07083, USA; 2 Department of Geography, University of Wisconsin-Madison, Wisconsin-Madison, WI, USA

**Keywords:** Tracking technology, Space-time activity, GPS, Individual behaviour, Influenza, Micro-scale, Disease transmission

## Abstract

**Background:**

Infectious diseases pose increasing threats to public health with increasing population density and more and more sophisticated social networks. While efforts continue in studying the large scale dissemination of contagious diseases, individual-based activity and behaviour study benefits not only disease transmission modelling but also the control, containment, and prevention decision making at the local scale. The potential for using tracking technologies to capture detailed space-time trajectories and model individual behaviour is increasing rapidly, as technological advances enable the manufacture of small, lightweight, highly sensitive, and affordable receivers and the routine use of location-aware devices has become widespread (e.g., smart cellular phones). The use of low-cost tracking devices in medical research has also been proved effective by more and more studies. This study describes the use of tracking devices to collect data of space-time trajectories and the spatiotemporal processing of such data to facilitate micro-scale flu transmission study. We also reports preliminary findings on activity patterns related to chances of influenza infection in a pilot study.

**Methods:**

Specifically, this study employed A-GPS tracking devices to collect data on a university campus. Spatiotemporal processing was conducted for data cleaning and segmentation. Processed data was validated with traditional activity diaries. The A-GPS data set was then used for visual explorations including density surface visualization and connection analysis to examine space-time activity patterns in relation to chances of influenza infection.

**Results:**

When compared to diary data, the segmented tracking data demonstrated to be an effective alternative and showed greater accuracies in time as well as the details of routes taken by participants. A comparison of space-time activity patterns between participants who caught seasonal influenza and those who did not revealed interesting patterns.

**Conclusions:**

This study proved that tracking technology an effective technique for obtaining data for micro-scale influenza transmission research. The findings revealed micro-scale transmission hotspots on a university campus and provided insights for local control and prevention strategies.

## Background

### Infectious disease transmission

The diffusion of a contagious disease traverses various scales from the local and regional to the global [1]. Mathematicians and geographers have long studied the spatiotemporal transmission of infectious diseases from different perspectives with numerical models [2-8], graph or mapping tools [9-11]. Recent outbreaks of highly communicable diseases [7,12-15] have triggered a marked rise in the number of studies on infectious disease transmissions [16], with equal amount of attention on the mechanisms of global disease spread [3,8,17], and at the regional scale of within countries or cities [4-7,13,18-21]. Only limited research, however, was reported at the micro scale, such as in the environment exemplified by a relatively closed campus, small community, school, residential, or hospital buildings with a few exceptions [22,23].

It is well recognized that human movement in the spatial and temporal dimensions has direct influence on disease transmission [18,24-27]. An infectious disease typically spreads via contact between infected and susceptible individuals in their overlapped activity spaces. Therefore, daily mobility-activity information can be used as an indicator to measure exposures to risk factors of infection. A major difficulty and thus the reason for paucity of studies of infectious disease transmission at the micro scale in the past was the lack of detailed individual mobility and exposure data. Since the records of small-scale movements and contacts between people were generally not available with only a few exceptions [22,23,28,29], studies of infectious disease were often aggregates in space and time [5].

### Tracking technology

Research on individual human space-time behaviour started first in the social sciences [30-36]. Previously in tourism research, transportation studies and shopping behaviour studies detailed space-time activity data often relied on the time-space diary technique, which requires subjects to actively record his or her activities in time and space. This method is highly demanding for the participants and collaboration from the participants greatly affects the quality of data [34,37].

Over the past decade, technology has transformed researchers’ ability to gather quantitative data on human activities. Global positioning systems (GPS), mobile communications, and wireless network make possible the tracking of human and object movements, generating large amounts of data with unprecedented quality and timeliness at relatively low cost. Applications of these technologies started to flourish in transportation research [38], retail studies [35], human ecology [36], and more recently, health related studies [27,39-54]. Examples of health related studies include investigating human mobility affected by diseases or surgeries or in young or aged populations [39-46], studying physical activity and the environment [48-51], and defining specific geographic contexts [52,53] or an operational “neighbourhood” in place-based health studies [54]. Kwan [52,53] has suggested that examining space-time activity spaces of individuals can lead to more accurate exposure measures. A couple of studies have attempted to track exposures to health risk factors related to human movement [27,47], with one targeting infectious disease but the focus was on vector-borne disease only.

The potential for using such tracking technologies is increasing rapidly, as technological advances enable the manufacture of small, lightweight, highly sensitive, and affordable receivers. The routine use of location-aware devices has become widespread (e.g., cellular phones) and regulations on the use of such technology (i.e. to protect user privacy mature [55]. The large amount of time-location data collected with these technologies allows for the investigation of space-time behaviour and its associated health impact [27]. This is especially relevant to the study of infectious disease transmission at the local to micro scales as precise structural details of the network of person-to-person contacts may be revealed.

A number of methods and technologies are currently available for tracking individual movements, among which GPS is the most commonly used in medical research because of its improving accuracy and portability. Compared to some controversial study that uses cell-phone data when the data collection and analysis was not disclosed to the users [36], GPS is also associated with less privacy issues as the participants are always informed about the study and only location information is collected. A number of studies have either evaluated the accuracy of portable, low-cost GPS devices [37,48,56,57] or have demonstrated with specific studies the feasibility and effectiveness of their uses in health-related research [39-46]. GPS, however, is limited to outdoor tracking. Indoor tracking technologies such as Radio frequency identification (RFID) and Wi-Fi-based positioning systems have only been used in modelling shopping behaviour in a specific grocery store [35] or tracking healthcare personnel or patients in a specific hospital [57] (and see [58] for a review) because all available indoor tracking systems are self-contained and require additional setup in the tracking environment, which can only be implemented within a fixed range.

Assisted GPS is one kind of the so-called hybrid positioning systems [42]. GPS uses radio signals from satellites for positioning. A-GPS uses not only satellite signals, but also cellular network resources to locate and use the satellites in poor signal conditions. Satellite signals can be weak due to dense buildings in an urban environment, tree canopy in a wooded area, or poor atmospheric conditions. In such case, A-GPS uses data from a cellular network to obtain faster fix times than standalone GPS. Fix time can be reduced from minutes to seconds and thus leads to more-accurate trip detection [49]. This is particular useful for tracking long durations when objects move both indoors and outdoors, as outdoor locations can be immediately picked up upon exiting from an indoor location.

This study uses A-GPS-based tracking devices to collect data of individuals’ space-time trajectories. One of the objectives of this study is to explore the potential of such technology to be used in the study of infectious disease transmission. To date, there are only a limited number of studies of human mobility in relation to disease transmission at the micro scale [27-29]. One of the studies used GPS to track human mobility [27] while the other two relied on traditional activity questionnaires [28,29]. The focus of these studies has been limited to vector-borne diseases. Vector-borne disease depends largely on spatial patterns of vector abundances and can thus be readily evaluated. In case of directly transmitted infectious disease, the risk is not known because the disease carriers engage in dynamic space-time activities themselves. Therefore, risk patterns could only be modelled by examining overlapping of space-time activity spaces of individuals and may possibly inferred by comparing the space-time behaviours of those infected with those of a control group during an outbreak. Therefore, daily mobility-activity information obtained from GPS tracking can be used as an indicator to measure exposures to risk factors of infection.

### Space-time data visualization and exploratory analysis

The space-time trajectory of human movement has many interacting dimensions: location, time, duration, sequencing and type of activities and/or trips [33,59]. Tracking devices can generate large volumes of mobility data in terms of time-stamped spatial locations. Such data is not only complex as they represent rough approximations of the complex human movement trajectories, but also semantically poor [60]. It is thus difficult to develop analysis techniques to extract meaningful abstractions from the raw data. Visualization has been suggested to be particularly suitable for dealing with such data when conventional inferential statistics and pattern recognition algorithms fail due to the complex attributes involved [33,59,61]. Exploratory visual analysis engages the powerful visual information processing and pattern recognition abilities of humans to reveal interesting patterns and lead to more focused and fruitful methods or models in later stages of a study.

Approaches to visualization and exploratory analysis of movement data include the use of static maps, animated maps, ringmaps [62] and space-time cubes (STC) [33,63-70]. Among these methods, the interactive 3D STC is the most endorsed because it is more intuitive than other 2D visualizations in handling the multi-dimensions of a space-time trajectory [71]. STC representation is based on Hagerstrand’s space-time model [72] and represents individuals’ movements in a cube where the base represents geography (space) and height represents time. It features geospatial lifelines related to the movement of individuals. While Miller [63,64], Neutens, Zeigler, and Schwanen [65,66] have focused on the accessibility problem induced by human mobility and focused on the conceptualization of and computation with space-time prisms, others [73] have focused on interaction among individuals during movement and suggested that STC as an effective tool in epidemiological research.

**Figure 1:**
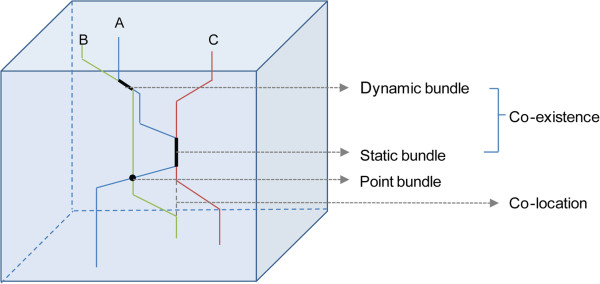
Space-Time Cube Representation of Modelled Student Activity and Interactions.

Figure 1 below illustrates a simplified STC representation of three college students’ activities in the morning of a typical school day, showing some of the concepts in time geography that are the most relevant to micro-scale disease transmission. It shows that student A and C sat in the same classroom for a class period (co-existence in terms of a static bundle). A and B walked by each other without more interaction (point co-existence) but met again later and walked along (co-existence in terms of a dynamic bundle). B and C never had direct interaction but the fact that C (and A) used the same classroom after B left from an earlier class makes it a situation called co-location. In the context of disease transmission, both dynamic bundles and static bundles are highly relevant, whereas point bundles and co-location beyond the lifespan of an airborne virus can be less important.

Ideally to model micro-scale disease transmission we need data on the inter-human interactions as illustrated in Figure 1. Not only outdoor, but also highly accurate indoor trajectories are needed for such purpose. Current technology and privacy concerns, however, still prohibit the collection of large amount of detailed trajectory data for modelling at this level. Therefore, although STC has been suggested as an effective tool in epidemiological research examples have been limited to large-scale dispersions of identified cases. In this pilot study, we collected detailed outdoor trajectories and inferred indoor stays through spatiotemporal processing of the data collected with an A-GPS. Approximated indoor stays cannot be as precise to specific locations inside a building. Thus we are not able to examine the actual human to human transmission patterns, but only rough estimates of risk patterns indicated by the overlapping of approximated space-time activity spaces of individuals. Risk patterns related to individual’s space-time behaviour, however, may be possibly inferred by comparing the behaviours of those infected with those of a control group during an outbreak.

One other issue regarding the use of STC for visual exploration of trajectory data is that large data volumes may result in apparent visual clutters that prohibit immediate visual detection of any patterns [59,60,67]. A few researchers have tackled the visual clutter problem with different approaches. Kwan [59] used density surfaces to represent the spatial distributions of activity intensities. High density indicates more overlapping of individuals’ activity spaces and more chances of human to human contacts that are relevant to the spread of flu viruses. Other methods used to support visual examination of large sets of movement data involve data aggregation [67,68,74]. Data is aggregated either on the spatial or temporal or both dimensions and clustering techniques are applied to extract generalized patterns.

This paper reports some initial attempts to analyze the trajectory data collected using A-GPS devices to examine space-time behaviours. Existing methods and others will be used to map space-time activity spaces and compare activity patterns of students who contracted the flu in contrast to those who were not infected. To summarize, this study aims for using A-GPS tracking to collect data, processing the data and visually exploring patterns for micro-scale disease transmission investigations. Potential findings may benefit micro-scale infectious disease transmission modelling and local prevention, control, and containment decision making by providing insights into specific questions such as:

1) How does one’s habit in terms of spending time in different places on a university campus affect chances of infection?

2) Are there certain places/buildings that lead to higher probability of infection?

3) Is a heavily trafficked cafeteria or student centre particularly susceptible to the spread of viruses and thus increase the chance of flu infection?

## Methods

### Data collection

**Figure 2:**
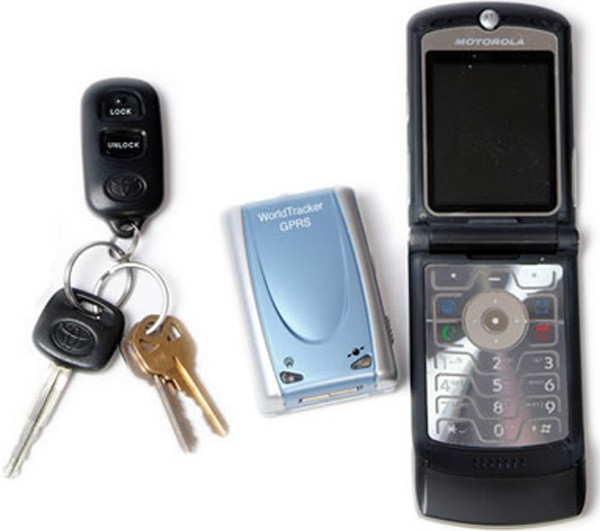
A-GPS device employed in this study.

The main tracking device used in this study is a commercial child tracker device, WorldTracker GPRS. It is highly portable (see Figure 2 for its size compared to a set of keys and a cell phone) and thus does not disrupt or affect one’s normal activity pattern once carrying it to log movements. It uses the SiRF Star III GPS Chipset with high sensitivity to enhance performance in low signal areas. It is A-GPS based with a fast fix time (in seconds) and accuracy within 3 meters in typical outdoor environments [75]. It does not store data in the device but transmits constantly the time and location information to a central storage server. Therefore participants do not need to perform any complicated operation except for charging it every night. One participant was also trained to carry a Garmin eTrex Venture HC GPS receiver and an Apple iPhone 3 with a tracking app (Path Tracker) installed at the same time to collect data with all three devices for one day for comparisons.

During the early spring of 2011 (the flu season), a total of 100 participants were recruited to carry the A-GPS device during their daily activities for a week. 96 of them generated over 400 valid day-trajectories. Ten students were also asked to actively record their stops and movements on an activity diary at the same time when they were carrying the devices. The diary contains columns about time, location, and activities such as taking class, dining, etc. for the student to fill up whenever he/she gets a chance to do so during the day. A three-day experiment in our study generated 30 trajectories paired with diary records.

Volunteers were recruited through the use of blast email and posters on campus. Enrolment is limited to full-time undergraduate student who did not receive flu vaccination for at least one year. Students were all interviewed on whether they have caught the flu during the current flu season, and if so, symptoms and severities were also recorded. Both recruitment and interviews were conducted based on protocols approved by the IRB of the University. No personal information was associated with the collected trajectories except flu status, gender and ethnical information.

### Data pre-processing and segmentation

Pre-processing is the cleaning of noisy raw trajectory data. Limited especially by the indoor positioning abilities of current tracking technology, trajectory data collected with all GPS devices tend to exhibit noises [52] especially during long period of stays in a building. Pre-processing is thus necessary as the first step of using the collected data. Previous research has addressed the problem with various filtering algorithms, such as though Gaussian Kernel smoothing or using a modified Kalman filter [49]. These filtering algorithms are general smoothing approaches used to reduce random errors in the data. Visual examination of the A-GPS trajectory data collected in our study reveals distinct patterns of errors resulting mostly from indoor stays. Such errors may be easily identified by overlaying the trajectory data with a detailed building layer and manually removed by selecting the erroneous track points. Large volume of data, however, prohibits manual processing. Therefore we developed both an interactive visual interface for manual pre-processing and a heuristic spatiotemporal algorithm based on the same mechanism for automatic batch processing [76].

**Figure 3:**
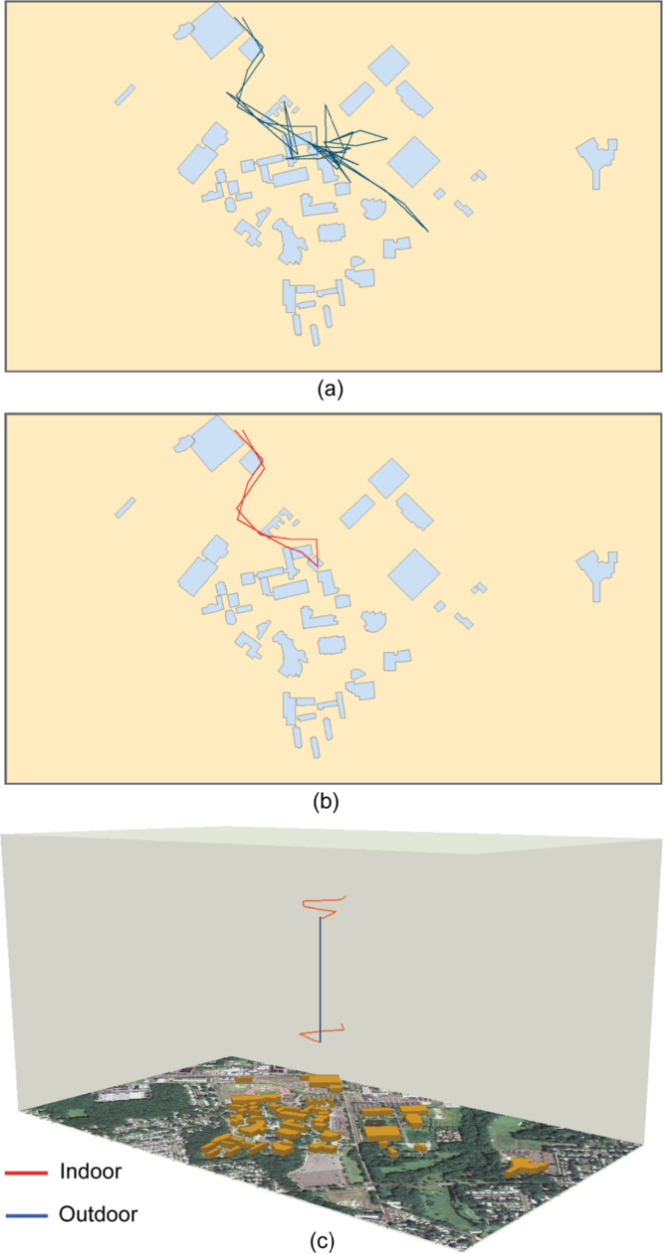
**Trajectory pre-processing and segmentation. (a)** a raw trajectory; **(b)** pre-processed trajectory; **(c)** pre-processed and segmented trajectory in a space-time cube.

The interactive option allows one to manipulate the 3D display of raw trajectories in the space-time cube representation. Noises may be identified based on the shape, speed and/or topology of track segments. Specifically, track points (vertices) with unrealistic high speed or abrupt direction change usually signify errors. A cluster of track points with spiky shapes (Figure 3a) spatially, located within and close to a building, and spanning a long duration temporally signifies positioning errors. This is because when signals are weak or absent, GPS locations are often off and appear to be toward random directions. Indoor segments thus tend to exhibit jagged or spiky shapes made up by many sudden directional changes in the trajectory. The interactive interface allows user to select a group of these points, calculate the spatiotemporal centroid of the selected points, and adjust the track to go through the centroid [76].

The automatic pre-processing algorithm searches through the loaded trajectory data and mimics the visual error detection approach mentioned above. Specifically, the algorithm starts from selecting segments with high roughness and then expands back and forth to clean up the trajectory. A spatial roughness indicator is used to reveal the spiky shapes representing noises resulted from indoor error. Other indicators such as speed and duration of the segment are also calculated. Obvious errors such as extremely high speed points are first removed. Spiky segments are then targeted to calculate their spatiotemporal centroids using a time-weighted cluster centroid approach. With this approach, tracking points that make up the spiky clusters are given different weights based on their “temporal importance”. Points that represent segments with longer durations are assigned higher importance. Once spatiotemporal centroids are identified, the trajectory is adjusted to pass through the centroid.

Trajectory segmentation involves the identification of indoor and outdoor parts as well as activities such as walking, driving from pre-processed space-time tracks. Our algorithm uses spatiotemporal centroids identified in the last step as seed indoor points and searches segments back and forth from the indoor centroid and assign those with very low speed indoor segments until speed starts to pick up. Besides criteria such as speed and duration of tracking points, spatial topology with regard to buildings is also used to label segments to corresponding categories. During this step, identified indoor segments are adjusted to geometries of the corresponding buildings. Activities such as attending a class, dining in the cafeteria, studying in the library, working out in the gym, etc. can be differentiated based on knowledge of the buildings’ usages.

### Space-time activity analyses and visualizations

The participants’ trajectories were used to model their space-time activities for the semester under study. The assumption was that individuals’ habitual behaviour in their activity space tends to repeat over time and thus tracking data sometime within the flu season could reveal useful information and patterns that are relevant to flu infections that happen in the season. This assumption is supported by recent research that models human mobility patterns concluding that humans are habitual animals whose space-time behaviours “follow simple reproducible patterns” [36] and that especially for university students activity patterns could be very similar from week to week during a particular semester as their class schedules are set.

The segmented trajectory data can be summarized to characterize one’s activity space. Attributes such as activity radius, average indoor stay per day, most frequently visited places, time spent in dormitory, cafeteria, classroom, gym, and so on may be easily derived. Such attributes can be analysed statistically to investigate their relationships to students’ flu status and also derive parameters to be used in local transmission models. This study, however, focuses on another perspective. With the availability of large amount of trajectory data, visual exploratory methods were applied to detect patterns.

(1) Density surfaces. The activity density pattern of the participants could be examined using density surface visualization as described in [33,59]. The density of different activities at different campus locations is visualized and the different patterns between the infected and non-infected may be examined. We developed a visualization tool that enables the mapping of three options of density surfaces [76]. With the first option, all vertices from the trajectory data are used to calculate kernel densities of the points. The second option calculates and displays density of individual paths travelled. And the third option re-samples the trajectory data using a set time interval and maps the densities of points spread evenly in time. This option is designed for tracking devices that collect tracking points in irregular time intervals due to varying sensitivity of the devices under various physical conditions or segmented trajectories, such as the child tracker we employed in our study. While density visualizations may help reveal overall patterns in the data, we could benefit from highlighting certain time periods to detect more detailed clusters and patterns. Temporal focusing [77] is a necessary addition to the control of a density surface visualization interface to allow for highlighting specific time periods and focusing on the selected sub-set of data for density surface calculation and visualization.

(2) Connection analysis. Connection analysis was conducted in our study in an attempt to identify strong connections among places of interests based on the trajectory data collected. For example, our study collected pedestrian trajectories on a university campus. Students walk to different buildings for taking classes, dining, and other purposes. A connection is a link represented by one or multiple segments in the collected trajectory data indicating a student has travelled from one building to another. Such connections can be derived from segmented trajectory data and the strength of each connection can be also determined by the volume of traffic that populates it. A connection analysis may help identify popular connections among campus buildings and infer typical activity sequences on campus. Hotspots such as those buildings with the most outbound or inbound traffic and hubs that connect the most trafficked places by a certain group of students (such as those who were infected by flu) may also be identified.

## Results and discussion

### Tracking data collection and pre-processing

A raw trajectory obtained from a student recording half day of his activity on campus using one of our A-GPS devices is illustrated in Figure 3a. The trajectory is displayed in 2D thus the time dimension is not shown. The campus buildings are shown as the spatial reference. It is noted that some portion of the trajectory close to a campus building appears to be noisy (indicated by the spiky portion of the track). This is caused by weak GPS signals around and inside buildings. The spike can sometimes reach very far due to the low-accuracy of positioning when the device is indoors. We employed the automatic pre-processing algorithm on the raw data and conducted spatiotemporal segmentation. Figure 3b shows the pre-processed trajectory and Figure 3c displays the segmented trajectory with color-coded indoor and outdoor segments in the space-time cube with reference to photo imagery and campus buildings. The horizontal dimensions represent space and the vertical dimension is time. While oblique lines indicate movement in space, straight segments should represent stays over a period of time.

**Figure 4:**
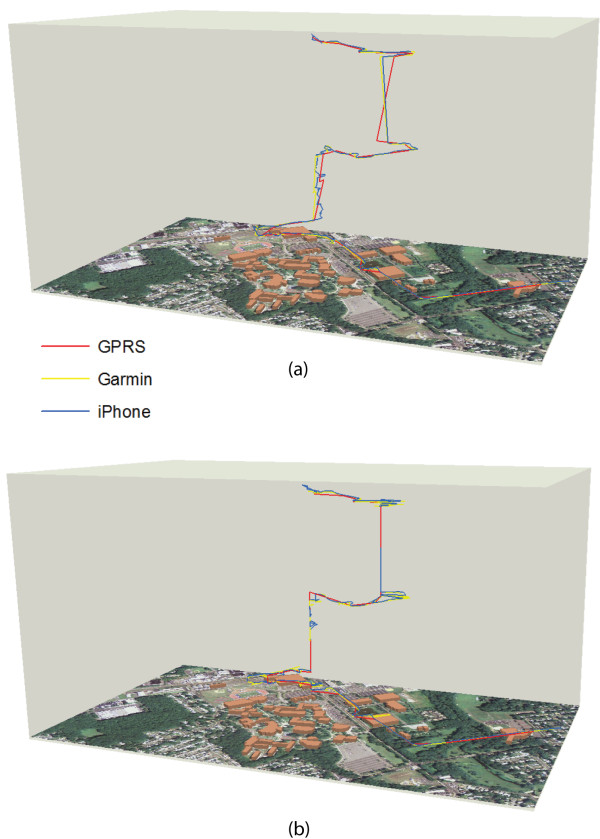
**Trajectories collected with an iPhone, a Garmin GPS, and an A-GPS based child tracker. (a)** raw trajectories; **(b)** pre-processed trajectories.

Trajectories collected by one participant using three different tracking devices are shown in Figure 4a. It shows that the three trajectories miss-match each other for some portions of the activity space, especially during indoor stays. Figure 4b shows the pre-processed trajectories and we see the three trajectories match each other much better after removing erroneous points. Table 1 lists the basic parameters of the three devices. The handheld GPS has the minimal time interval setting and longest battery life. But continuous data collection over several days would require participants to possess basic skills of using the GPS (i.e., saving tracks). iPhone 3 G has the shortest battery life when set to actively record and save locations. The A-GPS device we used, the Worldtracker GPRS, has a 15 hour battery life that enables the recording of an entire daytime trajectory with a simple plug-in charging at night. It also is not limited in data storage as the data transmits to the data server immediately via a cellular network. All three devices were reported to have similar spatial accuracies. The A-GPS, however, has the coarsest temporal resolution (15 seconds) among the three devices tested. These have resulted slightly more generalized trajectories in time-space as indicated by the red trajectory in Figure 4b.

**Table 1:** Basic parameters of the three tracking devices employed in the experiment

**Device**	**Minimum interval**	**Battery life**	Accuracy*	**Data storage**
**Handheld GPS** (Garmin eTrex Venture HC)	2”	22 h	3 m	In device
**iPhone 3** with Path tracker App	4”	4 h	<3 m	In device
**A-GPS** (WorldTracker GPRS)	15”	15 h	10-30 ft	Real time transmission

* Garmin and A-GPS accuracies are based on manufacturer specifications while iPhone accuracy is user-reported from online forums.

A three-day experiment was conducted with ten students to collect trajectories paired with diary records. Among the 30 trajectory-diary pairs, ten were excluded from our analysis as they either contain incomplete diary records (missing time periods in between records), very short or even no on-campus stays (less than 2 hours), or missing A-GPS signal (one A-GPS was out of battery in the middle of a recording due to forgotten charging). From the diary data, a total number of 55 indoor stays on campus with a total length of 63.15 hours were recorded. The pre-processed and segmented A-GPS trajectories captured 57 indoor stays with a total length of 65.40 hours. Two indoor stays described in the diary were missing from the trajectory data, while the later captured 4 more segments that were not recorded in the diary. The mismatch between the two sets of data could be due to errors in either the segmented trajectory data or the diary data or both. By examining the mismatches and interviewing students who participated in the study we found that one of the two “miss”es by the trajectory data lasted only 2 minutes and the other happened after a long-stay in a dorm building when the device probably has suffered from a slow fix time. It has also occurred to us that the diary takers may not have often recorded activities as soon as they happened but wrote their diaries out of memory when time allowed. As a result they often recorded down a rough estimate of time and some activities could be completely missing as such skipped their memories.

One problem we noticed from the comparison was that the segmentation algorithm we used sometimes mislabelled an indoor segment in a wrong building, especially when two buildings are connected to each other, which is the case with some buildings in our study, such as a group of dormitory halls on campus. Improvement on this aspect of the algorithm is needed. At the same time, an attempt to remediate the problem exercised in this study was to examine student behaviour by deriving attributes associated with a particular type of activity instead of the specific location-activity. The time that students spent in their dorm buildings is such an attribute. The dorm time recorded in the diary data totalled to be 10.70 and that derived from the trajectory data was 10.32 hours.

These two experiments indicate that all three commonly used GPS or A-GPS devices perform comparable in obtaining detailed trajectory data for space time activity studies. Handheld GPS supports the most temporal details but needs intervention for long durations of data collection. iPhone and the Worldtracker GPRS are both A-GPS based devices. However, the added indoor tracking ability does not lead to improvement as the cellular network positioning accuracy could be 50 meters or greater depending on the density of available cellular network. The battery life of the iPhone device is of the most concern in this experiment but new generations of the smart phone and better apps could improve significantly on this aspect. The Worldtracker GPRS employed in this study generates the coarsest trajectories but is the most user-friendly that doesn’t require any user intervention except for night charging. It also supports long duration of tracking as there is no limit of data storage. Comparison to diary data indicated that the trajectory data captured with the GPRS was able to approximate major patterns in students’ on-campus activities, and the 15 second time-interval still provides much finer temporal resolution than the traditional diary data could record.

### Exploratory analysis and visualizations

**Figure 5:**
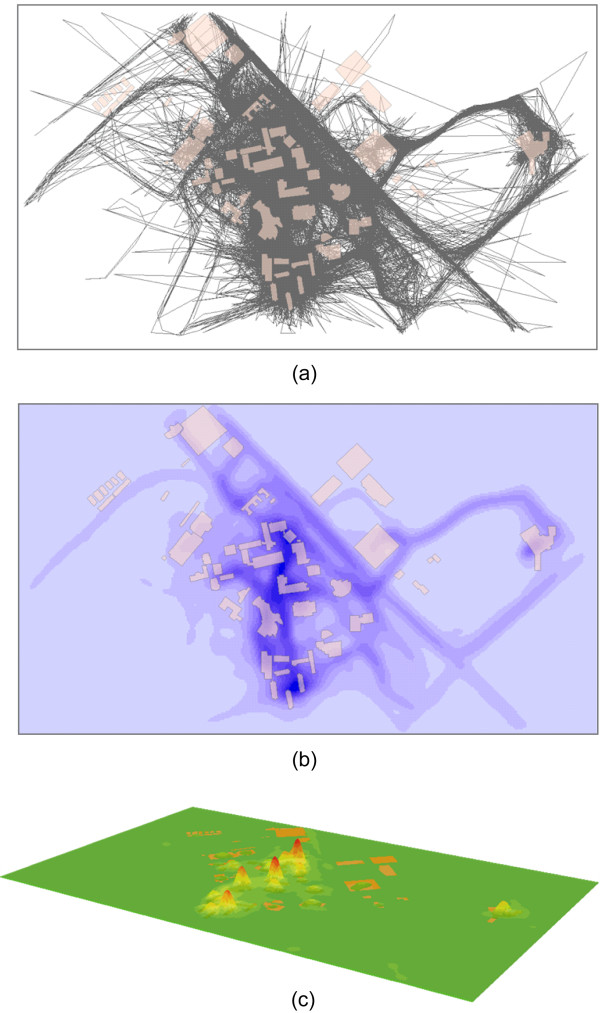
**Trajectory visualizations. (a)** original trajectories; **(b)** path density visualization in 2D; **(c)** 3D density surface visualization.

**Figure 6:**
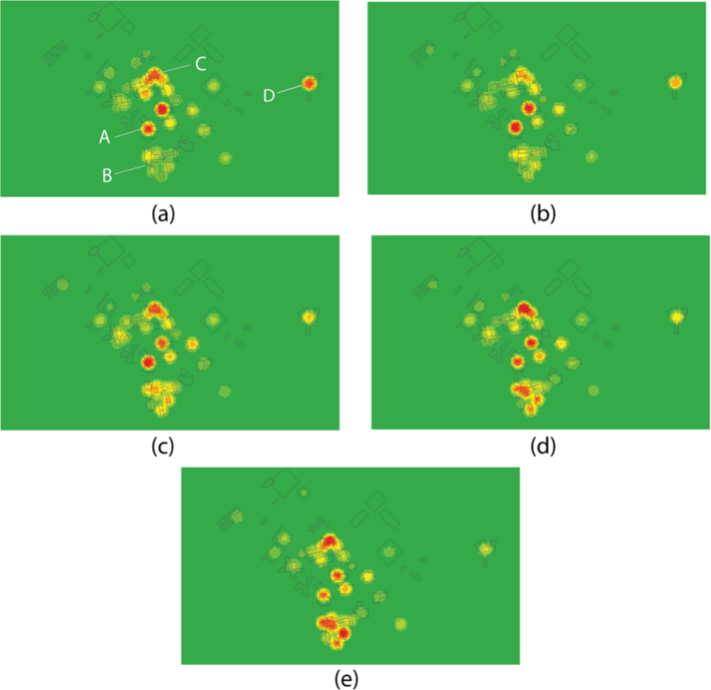
**Temporal focusing: density surfaces at different times of a day. (a)** morning; **(b)** noon; **(c)** early afternoon; **(d)** late afternoon **(e)** evening.

Figure 5a displays a total of 470 trajectories collected in our study. The visual clutter problem mentioned before is apparent when large amount of trajectories is involved. Figure 5b shows the density of the travelled paths on campus, from which it is observable that a few sidewalks are the most heavily travelled by the participants. Figure 5c shows the 3D density surface of space-time activities on campus, where it is notable that student activities are clustered around a few hotspots, including the University Center (UC), a group of departmental buildings, and residential halls. By controlling the time variable in the trajectory data through temporal focusing, we were able to examine spatial patterns at different time periods. Figure 6 shows the density surfaces at different times of a day in 2D, with red colour indicating high density and yellow being low. It is observed that activity patterns differ and hotspots may change throughout the day. For example, the UC (A) appears to be a hotspot from morning (Figure 6a) to late afternoon (Figure 6d) but slightly cools down toward the evening (Figure 6e). The dorm buildings (B) do not show high density until the late afternoon. The departmental building cluster indicated by label C seems to be crowded throughout the day but especially so in late afternoon and evening. The graduate college on east campus (D), however, only seem to be accommodating the most visitors in the morning.

**Figure 7:**
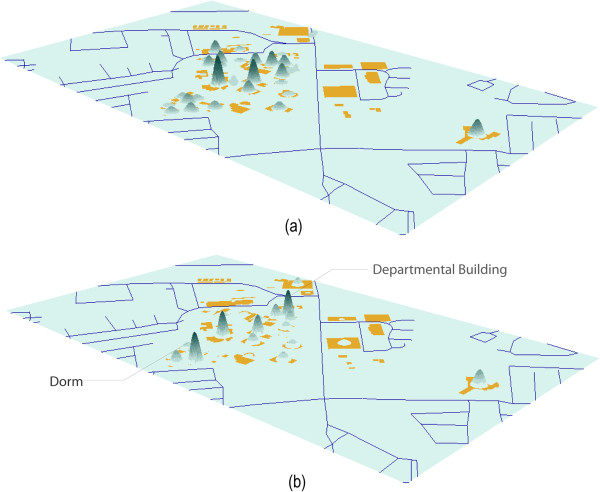
**Activity density patterns of two groups of students. (a)** students with were sick during the flu season; **(b)** students who were not sick during the flu season.

Comparing the density surface patterns of the infected group to the non-infected group (Figure 7), we see that there are less activity clusters for students who were sick in the season (Figure 7b) than those who were not (Figure 7a). Densities are also found to be slightly higher at the dormitory and at the departmental building clusters as shown at locations B and C in Figure 6. The departmental building cluster is home to three departments in the college of natural sciences that lie very close to each other and are connected by indoor pathways, although in separate buildings. Some preliminary speculations on the cause of this pattern include that on one side, the science students who take classes in these buildings tend to stay indoors for a long period of time. On another side, deriving from the temporal hotspot patterns in Figure 6, these buildings tend to be crowded especially in late afternoon or evening. The university class schedule indicates that classes scheduled in this time are often double periods-taking as long as 2 hour 45 minutes, leading to, again, prolonged indoor stay time. Further investigation, however, needs to be conducted to determine the exact causes of such clustering. This experiment indicates that the method has the potential to reveal hidden space-time activity patterns that may give insights to flu transmission at the micro-scale.

**Figure 8:**
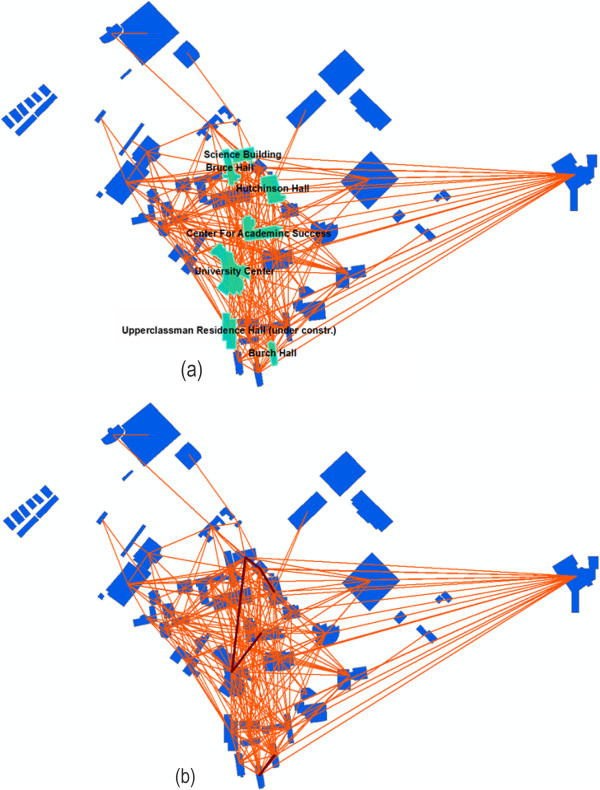
**Connection analyses for all participants. (a)** buildings highlighted indicating heavy traffic; **(b)** connections highlighted indicating heavy traffic.

**Figure 9:**
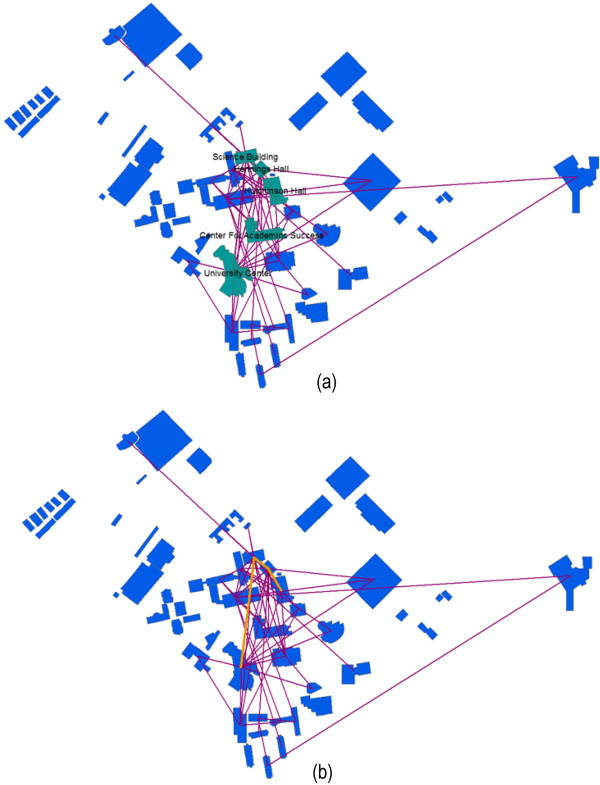
**Connection analyses for students who were sick during the flu season. (a)** buildings highlighted indicating heavy traffic; **(b)** connections highlighted indicating heavy traffic.

In addition to detecting specific space-time hot spots, connection analysis was conducted to identify strong connections among campus buildings based on trajectory data (Figure 8). Figure 8a shows all straight line connections among buildings captured by the participating students and the highlighted buildings are those with the highest outbound traffic volumes. Figure 8b illustrates the most trafficked connections (bold lines). It shows strong connections among the same group of departmental buildings identified as hotspots in density surface visualization. Strong connections also exist between this group of departmental buildings (with Science building being the centre) and the UC, and between the UC and the Center of Academic Success (CAS), a building that has undergraduate advisement offices and classrooms. Figure 9 shows the connection analysis results for only students who had been sick. Comparing Figure 9 to Figure 8, we see that one strong connection (between the UC and the CAS building) is no longer present from the set of strong connections identified for sick students only. The two strong connections that remain are one between the UC and the departmental buildings and another among the departmental buildings. The UC is the most heavily trafficked stop on campus being the main recreation centre where the cafeteria, book store, and recreation rooms are located. It was hypothesized as a potential high risk hub during flu season when students interact with each for a long period of time in a crowded space. The departmental buildings involved in the second connection are all attached to each other with indoor pathways. As indicated earlier, our segmentation algorithm has limitations when it comes to labelling segments with buildings especially when the buildings are close together or connected. Mislabelling may have contributed to part of the strong connection shown here. It is also speculated that these buildings have classrooms where students may spend many hours indoors taking classes without having to go outside of a building. These buildings are also relatively old constructions with aged ventilation systems that could increase risks of respiratory disease transmission. The CAS building that appears in the connection in Figure 8 but not in Figure 9, on the other hand, is a brand new building and stands by itself in a large open space. New ventilation and the fact that student activity has to often involve outdoor time periods whenever taking other classes out of the building both could lead to lower risks. These, are of course speculations but proves that such analysis, like other methods presented in this paper can be a useful exploratory analysis tool to reveal hidden patterns, given the availability of the detailed trajectory data.

## Conclusions and future work

Infectious diseases such as influenza remain an important global problem in public health. While efforts continue in studying the large scale dissemination of such highly contagious diseases, human behavioural modelling at the micro scale benefits local control, containment, and prevention decisions. This study investigated tools to collect data for the analysis of individual space-time behaviours and exploration of activity patterns.

Current tracking technology proved to be able to collect data accurate enough for space-time activity study in the micro-scale. Compared to the traditional activity dairy and questionnaire techniques, employment of GPS based devices, such as the A-GPS we experimented in this study, supports convenient collection of large amount of trajectory data. Once processed with an effective data cleaning algorithm the trajectory information can be used in various spatiotemporal analyses and exploratory visualizations such as the exercises discussed in this paper. Such analyses may help generate hypothesis on where and when people engage in behaviour which puts them at risk of contracting the flu through human-to-human contact or air inhalation in a relatively closed environment. In addition to the usual health advice like “wash your hands or avoid crowding”, more specific space-time activity recommendations could be made.

One limitation of our approach is that the segmented trajectory data sometimes mislabel an indoor segment in a wrong building, especially when two buildings are connected to each other, which is the case with some buildings in our experiment. Improvement on this aspect of the algorithm is needed. This study also only took some first steps into the examination of spatial patterns using the trajectory data collected. Following these steps, other methods like statistical analyses of variables characterizing one’s activity space and activity sequence analyses such as sequence alignment [41,78] can be performed in order to answer additional questions like how one’s activity space and sequences affect risks to infectious diseases. Spatiotemporal interactions such as bundles [64] may also be modelled to examine disease transmission patterns.

Despite the applicability of tracking technology in the study of disease risks at the micro-scale, readily available tracking technologies nowadays, however, still face the challenge of unsatisfactory accuracy for indoor positioning. Technologies such as RFID are limited in application scope as the tracking system is self-contained and require meticulous setup in the targeted environment [79]. Wi-Fi-based positioning can be a promising technology to lead to highly detailed space-time behaviour data collection and studies with much improved indoor accuracies. However, such high accuracy leads to more serious privacy concerns that may be another barrier for their uses.

Privacy has long been a major concern in both medical research and geospatial studies that involve tracking of individuals [80]. This concern has been one of the limiting factors in efforts to model infectious disease transmission at the micro scale. As need for real-time surveillance and intervention at the micro scale becomes more obvious with increasing threats of pandemics in a globalized world, and as the potential for precise surveillance and intervention explodes with the fast advancement of technology, it is critical to confront the privacy issue involved in using such technology. It is the social responsibility of researchers to protect privacy when using data such as the tracking trajectories collected in this study. Legal regulations regarding the use of such technology and data are also expected to be put in place for both research and practice. Although our current legal framework has not yet adapted to the potential privacy abuses of tracking technology [55], initiatives such as the US. Federal Communications Commission’s E-911 mandate has generated visions in the direction of progress in legal regulations regarding the use of such data and technology.

This study explores the potential of using tracking technology for research in infectious disease transmission and expects more applications when new, more accurate data is coming in no time with the current pace of technological advancement and privacy solutions or regulations set in place. With accurate indoor tracking technology, it is possible to set up real-time control and alert systems [79]. The idea is to monitor individual movements with portable devices during an outbreak. When someone is diagnosed as infected, his/her movements over the previous few days will be retrieved and others who have crossed paths with the infected will be given alerts to get checked. With more accurate indoor tracking, we are also able to obtain trajectory data in a single office or school building (such as the St. Francis school that marked one of the first outbreak of H1N1 flu infections in the US or a hospital ward that had many doctor infections in the Severe acute respiratory syndrome (SARS) outbreak in Hong Kong) to reveal indoor transmission patterns and even identify building ventilation problems.

## Abbreviations

GPS: Global positioning system; A-GPS: Assisted GPS; RFID: Radio frequency identification; SARS: Severe acute respiratory syndrome.

## Competing interests

The authors declare that they have no competing interest.

## Authors’ contributions

FQ contributed to the conception and the design of the study, collected the field data and contributed to the analysis and interpretation of the data and wrote the manuscript. FD contributed to the study design, the data processing, algorithm development, and visualizations. All authors read and approved the final manuscript.
